# Thermal, Hyperspectral, and Laser Doppler Imaging: Non-Invasive Tools for Detection of The Deep Inferior Epigastric Artery Perforators—A Prospective Comparison Study

**DOI:** 10.3390/jpm11101005

**Published:** 2021-10-05

**Authors:** Sebastian P. Nischwitz, Hanna Luze, Marlies Schellnegger, Simon J. Gatterer, Alexandru-Cristian Tuca, Raimund Winter, Lars-Peter Kamolz

**Affiliations:** 1 Division of Plastic, Aesthetic and Reconstructive Surgery, Department of Surgery, Medical University of Graz, 8036 Graz, Austria; hanna.luze@joanneum.at (H.L.); alexandru.tuca@medunigraz.at (A.-C.T.); r.winter@medunigraz.at (R.W.); lars.kamolz@medunigraz.at (L.-P.K.); 2 COREMED—Cooperative Centre for Regenerative Medicine, JOANNEUM RESEARCH Forschungsgesellschaft mbH, 8010 Graz, Austria; marlies.schellnegger@joanneum.at; 3 Division of Macroscopic and Clinical Anatomy, Medical University of Graz, 8036 Graz, Austria; 4 Medical University of Graz, 8036 Graz, Austria; simon.gatterer@stud.medunigraz.at

**Keywords:** perforator flaps, flap imaging, microsurgery, DIEP, thermal imaging, hyperspectral imaging, laser Doppler

## Abstract

Perforator flaps have become one of the leading procedures in microsurgical tissue transfer. Individual defects require a tailored approach to guarantee the most effective treatment. A thorough understanding of the individual vascular anatomy and the location of prominent perforators is of utmost importance and usually requires invasive angiography or at least acoustic Doppler exploration. In this study, we aimed at evaluating different non-invasive imaging modalities as possible alternatives for perforator location detection. After a cooling phase, we performed thermal, hyperspectral and Laser Doppler imaging and visually evaluated a possible detection of the perforator for a period of five minutes with an image taken every minute. We identified the most prominent perforator of the deep inferior epigastric artery by handheld acoustic Doppler in 18 patients. The detected perforator locations were then correlated. Eighteen participants were assessed with six images each per imaging method. We could show a positive match for 94.44%, 38.89%, and 0% of patients and 92.59%, 25.93%, and 0% of images for the methods respectively compared to the handheld acoustic Doppler. Sex, age, abdominal girth, and BMI showed no correlation with a possible visual detection of the perforator in the images. Therefore, thermal imaging can yield valuable supporting data in the individualized procedure planning. Future larger cohort studies are required to better assess the full potential of modern handheld thermal imaging devices.

## 1. Introduction

Skin defects secondary to chronic wounds, traumatic or oncological conditions require effective closure. Depending on the patient’s prerequisites and the extent of the defect and the involved tissues, a tailored approach needs to be derived for every such defect. One of the main techniques of reconstruction is the use of perforator flaps which have gained tremendous popularity in recent decades and were adopted into widespread fields of application throughout plastic and reconstructive surgery. A perforator flap is used for microsurgical tissue transfer of tissue(s) that are supplied by a single perforating artery and concomitant veins that derive from a deep vascular system and pierce (perforate) the underlying fascia or muscle. One of the most viable perforator flaps is the deep inferior epigastric perforator (DIEP) flap, which is located on the lower abdomen and mostly used for breast reconstruction due to its relatively voluminous constitution [1]. A profound understanding of the individual vascular anatomy is a necessary requirement for proper surgery planning, optimal surgical procedures, and successful outcome [2,3]. Despite known anatomical landmarks, individual features often necessitate preoperative imaging, especially with the DIEP flap, given its branched vascular anatomy and the often-pronounced thickness of the adipose layer [4,5,6]. Particularly the use of delicate perforators of a few millimeters’ diameter calls for a diligent individualized approach; insufficient understanding of the precise location of a perforator may jeopardize the entire procedure [7]. Perforator mapping has not only been demonstrated to increase safety, but to reduce morbidity, hospital length of stay and duration of surgery [5].

Depending on the respective institution, several perforator detection methods are in use, with CT- or MR-angiography (CT-A, MR-A) being the gold standard that could be complemented with new techniques [5,8,9,10,11]. Critics of these techniques describe several limitations thereof, e.g., radiation exposure (CT-A), need for an intravenous contrast agent, or imprecise transferability from a digital image rendered on a screen to the actual patient [12]. Generally accepted alternatives include duplex sonography or handheld acoustic Doppler [13,14,15]. In addition to CT-A, MR-A or duplex sonography, the preoperative perforator detection is frequently conducted using a handheld Doppler device at our institution. The handheld Doppler is preferred by many surgeons in clinical practice, given its widespread availability, its portability and low cost, while being able to locate perforators pre-, intra- and postoperatively [16]. It is straightforward to use and said to be the most commonly used perforator detection device [14]. Yet, some authors doubt its performance [17]: Like the color-coded Doppler ultrasound, the handheld Doppler requires training and is highly user-dependent, leading to reduced reliability, especially in small caliber vessels [18,19,20]. Moreover, its feedback is solely provided by an acoustic signal rather than a visible image that could indicate caliber and spatial orientation of the vessel. According to our own and the experience of other institutions, the handheld Doppler is a reliable tool for perforator detection with appropriate training [21]. Yet, a non-invasive, user-independent device with direct spatial correlation to the patient would be a valuable addition to the armamentarium of plastic and reconstructive surgeons.

The recent, diversified use of modern imaging devices for perforator detection and variable availabilities of devices [22,23,24,25] triggered the idea of comparing mobile smartphone-based thermal imaging (TI), hyperspectral imaging (HS) and Laser Doppler (LD) after a brief application of a cooling pack to the handheld Doppler. TI has been described as a quick and easy method warranting further investigation to assess thermal perforators in previous studies [9]. HS is a more recent technology assessing perfusion and oxygenation of tissues in different skin depths (up to 6 mm) [26,27]. This technique allows differentiation between better and lesser perfused areas, allowing for the indirect localization of a perforator [20]. LD is used routinely in burn surgery to assess tissue perfusion and, therefore, burn depth [28]. Furthermore, it is used to assess the perfusion of different perforator flaps [29].

This study’s aim was to increase evidence and to identify the most suitable non-invasive, portable imaging device for an individualized perforator identification approach.

## 2. Materials and Methods

The study was approved by the ethics committee at the Medical University Graz, Austria (31-477 ex 18/19). Written informed consent was obtained from all individual participants included in the study. All procedures performed were in accordance with the ethical standards of the institutional and/or national research committee and with the 1964 Helsinki declaration and its later amendments.

### 2.1. Detection Devices

TI: Non-invasive TI was performed with the FLIR One^®^ Pro for iOS Attachment for iOS Smartphones. (FLIR Systems, Inc., Wilsonville, OR, USA). Temperature differences of up to 0.07 °C can be detected by this device, enabling a distinction between the blood flow in the perforator and the surrounding “cooled” tissue. Detailed specifications are available on the manufacturer’s homepage [30]. Imaging acquisition was further processed with the FLIR One^®^ App.

HS: The TIVITA^®^ Wound (500–1000nm, Diaspective Vision GmbH, Am Salzhaff-Pepelow, Germany) was used for HS imaging. This technology uses the remission of light of different wavelengths of illuminated tissues to assess parameters like perfusion, oxygen saturation, and hemoglobin content. There is no direct contact between the device and the patient, and the results are visualized by color-coded images on a computer. Detailed specifications of the device are available on the manufacturer’s homepage [31].

LD: LD is a non-invasive imaging technique whose working principle is the Doppler shift of laser light that is backscattered by moving red blood cells in the cutaneous microcirculation [32]. The detection of the Doppler shift by optical heterodyning allows for rendering of an image, in a technique that can be considered analogous to the acoustic Doppler [33]. The moorLDLS-BI LaserDoppler (785 nm, Moor Instruments Ltd., Axminster UK) was used for LD imaging, and detailed specifications are available on the manufacturer’s homepage [34].

### 2.2. Study Design & Patient Collective

The study was designed as a prospective, monocentric comparison study. The study collective consisted of 18 patients that were treated for any reason requiring inpatient treatment at our institution (Division of Plastic, Aesthetic & Reconstructive Surgery). Inclusion criteria were: (1) inpatient stay at our department, (2) individual willing to participate in this study, (3) between 18 and 80 years old. Patients that (1) had any prior surgery in their abdominal region, (2) showed scars on their abdomen, (3) suffered from any known disease compromising micro-circulation (like diabetes mellitus or peripheral arterial obliterative disease) or (4) received steroid or other immunosuppressive therapy were excluded from the study. We chose to further exclude patients with planned DIEP surgery to not increase burden on those patients.

The patient collective of 18 patients was divided into three groups with six patients each. Each participant’s abdominal area was investigated with each of the three devices, with the group determining the sequence of the different acquisition methods to avoid possible bias by repeated measurements. Group 1’s order was TI, LD, HS; group 2’s order was HS, TI, LD; group 3’s order was LD, HS, TI. The group allocation was chosen merely on patients’ age to reach a homogeneous age distribution amongst the groups. Due to the low case numbers, we chose not to randomize patients.

### 2.3. Study Course

Prior to any study-related activity, the participants were informed in detail, and written informed consent was obtained. Sex, age, Body-Mass-Index (BMI) and abdominal girth (rounded to the full cm) were assessed. The region of interest (ROI) was defined as a square of 10 × 10 cm spanning from the umbilicus downwards around the midline. This ROI was chosen based on clinical experience and easy reproducibility. The ROI was identified and screened for a perforator with the acoustic handheld Doppler.

**Figure 1:**
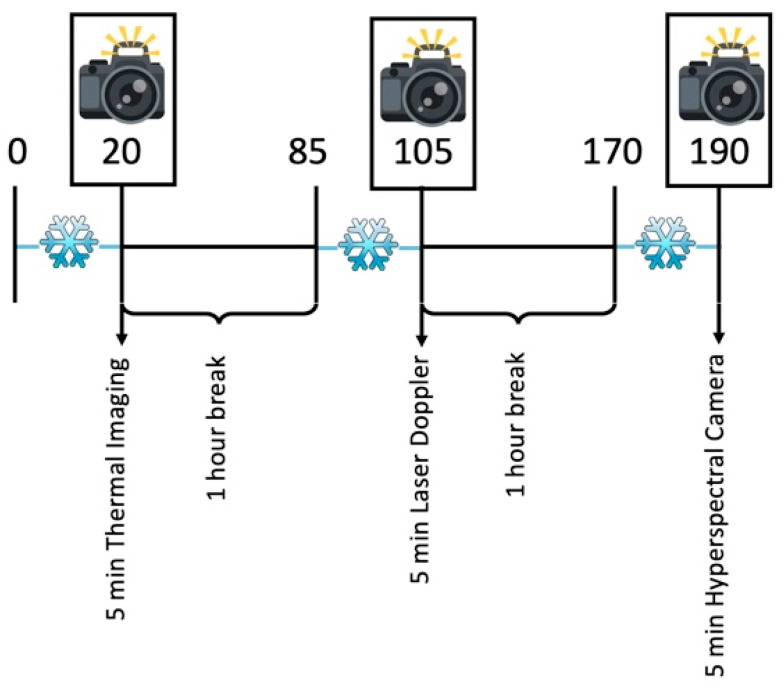
Exemplary study course for group 1. After 20 min of cooling, thermal imaging was performed minutely for five minutes. After an hour break the same process was repeated with laser Doppler and hyperspectral imaging after another hour break.

The ROI was cooled for 20 min with a commercially available cool pack that had been stored in a temperature-monitored fridge at 5 °C for at least 20 h, to diminish perfusion of the abdominal skin. The cool pack was then removed and the respective image acquisition method was employed every 60 s for a total of 5 min to evaluate changes in detection rate over time since cessation of cooling. The subsequent image acquisition methods were employed following a break of 60 min in the same manner. We therefore acquired 6 images per patient per imaging method, resulting in 324 images overall. The study course for group 1 is depicted in Figure 1.

After completion of image acquisition, two investigators who are plastic surgery residents experienced in the use of the pencil Doppler, identified the most prominent perforator in the area independent of each other, which would most likely be chosen were a procedure to be conducted. If concordance could not be reached, both identified perforators were used as further reference. Perforators outside the ROI were not considered.

### 2.4. Image Analysis

The TI, HS and LD images were then screened for a possible perforator (“warmer” area in TI, “more perfused” are in HS and LD). The identification of a perforator location on TI, HS and LD images was conducted by two different investigators that were blinded to the pencil Doppler assessment. The identification was at the discretion of the investigators, given the lack of predefined requirements for a perforator in these image acquisition methods in the literature. The ROI in the images was divided into 16 equal squares (4 × 4). Then, perforator locations in TI, HS and LD images on one side, and the locations of pencil Doppler identification were compared and matched. A positive correlation (true positive) was assigned to the image if the perforator’s location in the ROI matched. Any image with a visible perforator that was distinctly more prominent than the reference perforator was declared false positive. Several visible perforators were seen as true positive, if at least one reference perforator was included as well. If no perforator was seen at all, pictures were classified as true negative (if no perforator had been detected by pencil Doppler) or false negative if a perforator had been identified by pencil Doppler. First, patients have been evaluated for their availability of a positive match, but ultimately, since the aim was to investigate a possible detection over time since cessation of cooling, images have been evaluated rather than patients.

### 2.5. Statistical Analysis

Normality testing was not performed given the small sample size and the expected low power thereof [35]. Data is presented using medians and 25th and 75th percentile. Specificity, sensitivity, wrong positive and negative rates were calculated using cross-tabulation. Differences between the groups were determined using One-Way ANOVA. Spearman correlation coefficient was used to determine correlation between biometric parameters and a successful match to investigate a possible bias. The level of significance was set to *p* < 0.05. Prism 9.0.2 (GraphPad Software, LLC., San Diego, CA, USA) was used for statistical analysis.

## 3. Results

A total of 18 patients were investigated in three different groups (group 1: subject 01-06, group 2: subject 07–12, group 3: subject 13–18). The groups showed no significant difference in sex, (*p* = 0.34), age (*p* = 0.76), BMI (*p* = 0.30) or abdominal girth (*p* = 0.27). The participants’ specifics are displayed in Table 1.

### 3.1. Perforator Detection

In all 18 participants, at least one perforator was detected by pencil Doppler. Nine participants showed perforators on the left side, three on the right, and six on both sides with two reference perforators.

**Figure 2:**
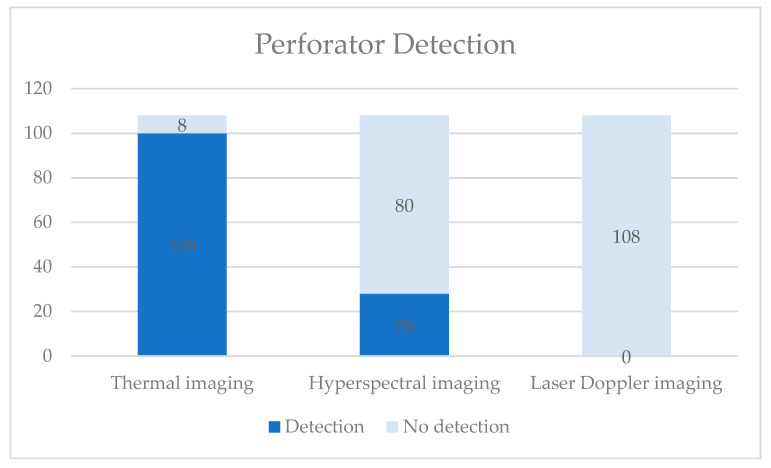
Summary of the detection results of the three imaging methods.

In total, 324 images were taken with TI, HS and LD (108 each). In 128 images, a match was observed (39.51%). One participant (5.56%) showed no match at all, independent of the imaging method. No correlation was seen between a possible match (true positive) and any of the parameters mentioned above (*p* > 0.05). There was no correlation between the imaging sequence (group) and a possible detection (*p* > 0.05). Table 2 and Figure 2 summarize the detection of the perforators.

### 3.2. TI Detection

**Figure 3:**
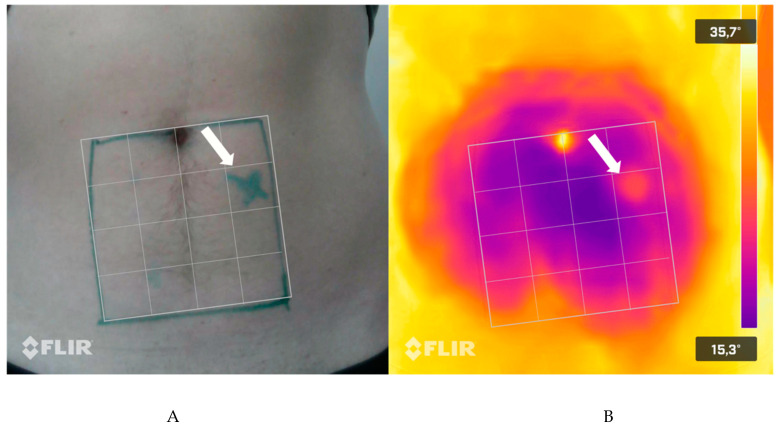
Exemplary depiction of a marked perforator (**A**) and the corresponding perforator in the TI (infrared) image (**B**).

On a patient level, 17 of 18 patients showed a positive match (true positive) resulting in a sensitivity of 94.44%. One-hundred of 108 images showed a matching perforator resulting in a sensitivity of 92.59%. All six images of 16 patients showed a positive match. One patient’s images started to match after two minutes and one patient’s images showed no perforator at all. The false negative rate is therefore 5.56 (1/18 patients) or 7.41% (8/108 images). No false positive (0%) perforator has been detected. Since no true negative could be detected (no patient without a perforator in the handheld Doppler), specificity cannot be indicated. The temporal aspect did not significantly influence the detection rate, since in all but one patient’s images the perforator could be seen immediately after the cooling as well as 5 min later. Figure 3 shows an exemplary TI image with a detected perforator.

### 3.3. HS Detection

**Figure 4:**
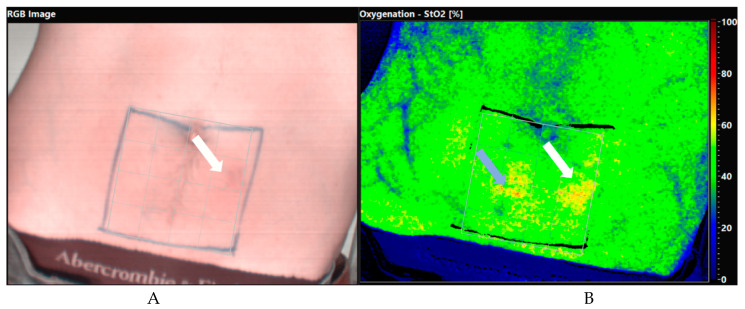
Exemplary depiction of a marked perforator (**A**) and the corresponding perforator in the HS (Oxygenation StO_2_) image (**B**). The mark has been removed and is visible palely to not interfere with the HS imaging. The grey arrow shows another possible perforator that had not been referenced with the pencil Doppler; it was not distinctly more prominent than the reference perforator.

On a patient level, 7 of 18 patients showed at least on positive match, resulting in a sensitivity of 38.89%. Twenty-eight of 108 images showed a matching perforator resulting in a sensitivity of 25.93%. Three patients’ images showed the reference perforator in all six images, in two patients the perforator was seen for two minutes (3/6 images) and in two patients for one minute (2/6 images). Eleven patients showed no matching perforator at all, yielding a false negative rate of 61.11% (11/18 patients) or 74.07% (80/108 images). No false positive perforator has been detected. Since no true negative had been detected (no patient without a perforator in the handheld Doppler), specificity cannot be indicated. Figure 4 shows an exemplary HS image with a matching perforator.

### 3.4. LD Detection

**Figure 5:**
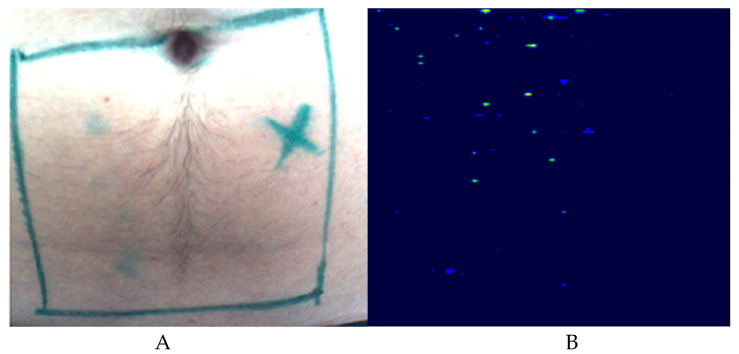
Exemplary depiction of a marked perforator (**A**) and the corresponding LD image (**B**), where no perforator was detectable.

None of the 108 images allowed the detection of the reference perforator yielding a sensitivity of 0% in this setup. Since no true negative has been detected (no patient without a perforator in the handheld Doppler), specificity cannot be indicated. Figure 5 shows an LD image of an abdomen, where no perforator was detected.

## 4. Discussion

Our study investigated the usability of non-invasive, portable imaging devices compared to the handheld Doppler to support an individualized approach in abdominal perforator detection as used in the DIEP flap. We could demonstrate a sensitivity of 94.44%, 38.89% and 0% for TI, HS and LD in this study setup respectively.

While all three methods are used in different clinical indications to assess skin perfusion to some extent, the acquired results yielded different outcomes: TI is a rather new technology indicating a surface’s temperature by a smartphone-compatible plug-in. In our study, we could reach a sensitivity of 94.44% when compared to the handheld pencil Doppler device. These results are very promising and quite similar to the ones obtained in other studies with a sensitivity of 86.2% and a specificity of 80% [36], but could not reach the sensitivity of 100% and the specificity of 98% postulated by Pereira et al. who compared the FLIR One to a CT angiography in 20 patients with 38 anterior-lateral-thigh (ALT) flap regions. These rates, however, strongly depend on the methods of evaluation and the standard compared to. In our study setup, image acquisition followed a 20 min period of cooling. This setup was chosen to show the dynamic reperfusion of the abdominal skin to identify the “competing” perforators as proposed by Weum et al. and Muntean et Achimas-Cadariu [37,38]. Other study groups have published TI perforator studies with limited (air fan on the bare abdomen for two minutes) [39] or no cooling at all [23], reaching a sensitivity of 100% and 67% respectively, compared to their standard (intraoperative visualization and pencil Doppler). Both study groups used an older TI device with different specifications. As indicated by de Weerd et al. [40] the “cold challenge” was not reported as uncomfortable by any patient in our study either, and might prove helpful in identifying a more efficient perforator having pushed through the cold earlier than the competitors. We could, however, not reach our goal of completely inhibiting the abdominal skin perfusion entirely using our cold challenge to visualize the dynamics of reperfusion, as perforators in all but one participant were seen immediately after removal of the ice pack. Therefore, our results show a high diagnostic value of the FLIR One TI, which at least complements the preoperative perforator imaging. We observed no correlation of sex, age, BMI, abdominal girth with a possible perforator detection, therefore implying a universal applicability in most patients. TI is a promising and individualized alternative in case CT angiography is unavailable or contraindicated (e.g., allergy to contrast agent) or a lack of experience with the handheld Doppler device to at least support the latter. However, future larger scale studies are necessary to substantiate these findings. TI can be manually calibrated prior to its use to adapt to the surrounding temperatures. Furthermore, it impressed with its ease-to-use requiring close to no training at all, while being cost efficient (currently retails at 229.99$ at the FLIR One homepage) and readily available. The fact that it is used in a no touch, non-invasive technique that can be performed basically anywhere additionally underscores its versatility. Amongst others, its medical applications are the evaluation of burn depth [41,42,43], detection of postoperative infections [44] or intra- or postoperative free flap monitoring [45,46], but it also allows the assessment of specific research interests [47].

The results of the HS imaging could not reach the accuracy of TI. A sensitivity of 38.89% on a patient level, and 25.93% on an image level is surprisingly low in contrast to the only other HS perforator detection study we could retrieve from the literature: Goetze et al. achieved a sensitivity of 97% when comparing HS to color-coded ultrasound in the detection of ALT flap sites [20]. In two of our study participants the detected perforator was seen for one, and in two participants for two minutes after the 20-min cooling period, before becoming indistinguishable from the surrounding tissue. Goetze et al. used a cooling protocol of three minutes with another three minutes of reperfusion without cooling, in which the images were acquired and showed the highest concordance after three minutes of cooling and after one minute of reperfusion. This might suggest that cooling is indeed necessary for a proper perforator detection, but the timing remains to be investigated. However, it appears that our cooling protocol might have been too long for most of the perforators to be detected. Other parameters like body temperature and blood pressure might also influence a detection and should be evaluated as confounding factors in future assessments. Possibly, another reason for these highly deviating results might be the fact that a different HS device was used, having other focal points within the included software package. Another downside we experienced compared to TI was the unwieldy design of the HS camera and the need for a dark surrounding environment. The HS camera requires a desktop computer and is therefore attached to a trolley, resulting in a bulky device, limiting ease of transport from A to B. Retailing at several thousand Euros, the device requires significant investment. While we could not reproduce the promising results of Goetze et al. in our study and are unable to recommend the device for perforator detection with the described cooling protocol, it has been demonstrated to be of significant value *following* surgical anastomosis for perfusion monitoring and to detect underperfused areas within the flap in other studies [48,49]. Some authors postulate it to be even more effective than the gold standard for flap monitoring, clinical assessment and handheld Doppler [50,51]. Similar aspects are valid for LD imaging. As we did not detect a single perforator using LD in our study, we deem it not suitable for perforator detection in conjunction with our cooling protocol. Significant asset cost, bulky device and training requirements are similarly valid for LD and for HS imaging devices. Just like HS, the value of LD does not lie in the detection of perforators, but the detection of perfusion changes, therefore being a valuable tool for flap monitoring *after* surgery [52,53,54].

### Limitations & Outlook

One of this study’s limitations is that we have assessed the perforators in a limited number of patients who were not scheduled for flap surgery; therefore, we had no visual confirmation of the perforator to serve as control. Furthermore, the low number of participants in the three groups could possibly conceal an influence of the repeated measurements and is a limitation for meaningful statistical analysis; due to the low case number, we also decided not to analyze the influence of co-morbidities and medication on possible detection. Since no true negatives and false positives were detected, the validation of the devices in our study is also limited. Our study population consisted of only 33.3% females; even though we did not demonstrate a correlation between gender and possible detection, DIEP flaps are usually performed in females. Hence, the gender distribution should be listed as a limitation as well. Another major limitation is the fact, that the detection of a perforator was at the subjective discretion of investigators. We were not able to retrieve predefined requirements for a perforator to be objectively evaluated as “positive” in these imaging methods from the literature. To reduce the impact of this limitation, we had two independent investigators assess the images who were blinded to the handheld Doppler. Additionally, a possible bias cannot be excluded due to human error. Another limitation is our study setup that included a 20-min cooling period prior to the imaging, which might have had an influence on the sensitivity of the devices. Physical parameters like blood pressure and body temperature, which we did not assess, should also be evaluated in future assessments to detect possible correlations. Since we defined a ROI below the umbilicus, it is possible to have missed matching perforators outside of the ROI, and of course, to have misinterpreted findings. Lastly, since the devices have their imaging focus in different depths (surface vs. mm), results should be considered complementary and not competitively.

Future studies should focus on different cooling strategies/protocols, and the question whether these are actually necessary for perforator detection with TI. They should be designed as prospective case-control-studies with larger patient cohorts.

## 5. Conclusions

In conclusion, we conducted this study to assess non-invasive, portable imaging tools as to their ability to detect perforators. We demonstrated a high value of TI for perforator detection, being almost as effective as the handheld Doppler device, which is in frequent use in our institution. To obtain the best possible outcome after surgery, an individualized approach should be derived. This approach should not only take into consideration the individual patient, but also the treating physician, institution and everyone else in patient care. Individual patients requiring a sophisticated reconstruction with a perforator flap should be planned as individually and personalized as possible. Therefore, perforator detection by TI can be a valid alternative in a patient with an allergy to contrast agents, or a surgeon not sufficiently experienced with the handheld Doppler. Postoperative flap monitoring could be performed by HS or LD, both of which are not as suitable for perforator detection, according to our results. A combination of available tools/devices should be used that is most appropriate, with each device being justified given the respective indication [33] to guarantee the most beneficial possible for the patient.

## Figures and Tables

**Table 1:** Participants’ characteristics. The order of the imaging was determined by the group. Group 1: Subject 01–06. Group 2: Subject 07–12. Group 3: Subject 13–18. BMI = Body Mass Index. m.d. = missing data.

Subject ID	Sex	Age [years]	BMI [kg/m^2^]	Abdominal Girth [cm]	Perforator Detection
01	Male	36	m.d.	m.d.	Left
02	Male	30	22.86	78	Right
03	Male	58	29.30	111	Right
04	Male	41	32.53	108	Left
05	Male	65	m.d.	m.d.	Left
06	Female	71	27.89	92	Left + Right
07	Female	28	23.88	82	Left + Right
08	Female	22	19.13	68	Left
09	Female	52	23.44	70	Left + Right
10	Male	41	31.56	102	Left
11	Male	61	22.21	92	Left + Right
12	Male	62	23.66	93	Left
13	Male	24	25.98	92	Left
14	Female	24	20.44	70	Left
15	Male	49	32.77	132	Right
16	Female	45	34.72	110	Left
17	Male	60	26.47	100	Left + Right
18	Male	60	25.08	98	Left + Right
Total/median (25th, 75th percentile)	66.6% Male 33.3% Female	47.00 (29.50, 60.25)	25.53 (23.01, 31.00)	92.50 (79.00, 106.50)	9 Left, 3 Right 6 Left + Right

**Table 2:** True positive images per patient. Group 1: Subject 01–06. Group 2: Subject 07–12. Group 3: Scheme 13. TI = Thermal Imaging, HS = Hyperspectral Imaging, LD = Laser Doppler Imaging.

Subject ID	TI	HS	LD
01	6	0	0
02	6	0	0
03	6	3/6	0
04	6	2/6	0
05	6	3/6	0
06	4/6	0	0
07	6	0	0
08	6	0	0
09	6	0	0
10	0	0	0
11	6	0	0
12	6	6	0
13	6	2/6	0
14	6	0	0
15	6	0	0
16	6	6	0
17	6	0	0
18	6	6	0
Total	100/108	28/108	0/108

## Data Availability

Detailed data supporting the results are available at the authors.
